# CD163^+^ macrophages in the triple-negative breast tumor microenvironment are associated with improved survival in the Women’s Circle of Health Study and the Women’s Circle of Health Follow-Up Study

**DOI:** 10.1186/s13058-024-01831-8

**Published:** 2024-05-08

**Authors:** Angela R. Omilian, Rikki Cannioto, Lucas Mendicino, Leighton Stein, Wiam Bshara, Bo Qin, Elisa V. Bandera, Nur Zeinomar, Scott I. Abrams, Chi-Chen Hong, Song Yao, Thaer Khoury, Christine B. Ambrosone

**Affiliations:** 1 Department of Cancer Prevention and Control, Roswell Park Comprehensive Cancer Center, Buffalo, NY USA; 2 Department of Pathology, Roswell Park Comprehensive Cancer Center, Buffalo, NY USA; 3 Department of Biostatistics and Epidemiology, Rutgers School of Public Health, Piscataway, NJ USA; 4 Cancer Epidemiology and Health Outcomes, Rutgers Cancer Institute of New Jersey, New Brunswick, NJ USA; 5 Department of Immunology, Roswell Park Comprehensive Cancer Center, Buffalo, NY USA

**Keywords:** Tumor-associated macrophages, CD163, Breast cancer, Prognosis, Survival

## Abstract

**Background:**

Tumor-associated macrophages (TAMs) are a prominent immune subpopulation in the tumor microenvironment that could potentially serve as therapeutic targets for breast cancer. Thus, it is important to characterize this cell population across different tumor subtypes including patterns of association with demographic and prognostic factors, and breast cancer outcomes.

**Methods:**

We investigated CD163^+^ macrophages in relation to clinicopathologic variables and breast cancer outcomes in the Women’s Circle of Health Study and Women’s Circle of Health Follow-up Study populations of predominantly Black women with breast cancer. We evaluated 611 invasive breast tumor samples (507 from Black women, 104 from White women) with immunohistochemical staining of tissue microarray slides followed by digital image analysis. Multivariable Cox proportional hazards models were used to estimate hazard ratios for overall survival (OS) and breast cancer-specific survival (BCSS) for 546 cases with available survival data (median follow-up time 9.68 years (IQR: 7.43–12.33).

**Results:**

Women with triple-negative breast cancer showed significantly improved OS in relation to increased levels of tumor-infiltrating CD163^+^ macrophages in age-adjusted (Q3 vs. Q1: HR = 0.36; 95% CI 0.16–0.83) and fully adjusted models (Q3 vs. Q1: HR = 0.30; 95% CI 0.12–0.73). A similar, but non-statistically significant, association was observed for BCSS. Macrophage infiltration in luminal and HER2+ tumors was not associated with OS or BCSS. In a multivariate regression model that adjusted for age, subtype, grade, and tumor size, there was no significant difference in CD163^+^ macrophage density between Black and White women (RR = 0.88; 95% CI 0.71–1.10).

**Conclusions:**

In contrast to previous studies, we observed that higher densities of CD163^+^ macrophages are independently associated with improved OS and BCSS in women with invasive triple-negative breast cancer.

*Trial registration*

Not applicable.

**Supplementary Information:**

The online version contains supplementary material available at 10.1186/s13058-024-01831-8.

## Background

The tumor-immune microenvironment (TIME) has a key role in pathologic complete response and patient survival in breast cancer [1–4]. While tumor-infiltrating lymphocytes (TILs) in aggregate and various T cell subpopulations have been routinely examined, tumor-associated macrophages (TAMs) and other cells of the myeloid lineage have received less attention, despite being a prevalent immune subpopulation in breast carcinoma. Typically, high macrophage counts in breast tumors are regarded as being associated with tumor progression and poorer survival [5–8]. However, much prior work on macrophage markers in relation to breast cancer outcomes had small study samples that precluded analyses stratified by subtype, or adequately powered analyses adjusted for prognostic factors that are known to influence breast cancer survival. Moreover, most of these earlier studies were overwhelmingly conducted in populations of White or Asian women, and representation of Black women on this topic is poor, with only a handful of studies to date [9–11].

Novel therapeutic approaches that target macrophages are an increasingly important area of clinical study, and thus it is important to understand how specific macrophage markers vary in accordance with demographic and clinical factors [12, 13]. As part of our ongoing work that investigates the breast TIME in relation to aggressive disease and poorer outcomes in Black women, we investigated the macrophage marker CD163 among women participating in the Women’s Circle of Health Study and Women’s Circle of Health Follow-up Study. Our objective was to compare macrophage infiltration between Black and White women and to investigate the association of CD163^+^ cells with overall and breast cancer-specific survival in a study sample that was large enough to allow stratification by subtype and adjustment for known prognostic factors in breast cancer.

## Methods

### Study population

We used data and tissue samples from women participating in the Women’s Circle of Health Study (WCHS), a multi-site, case–control study designed to evaluate the risk factors for aggressive breast cancer in Black and White women, and the Women’s Circle of Health Follow-up Study (WCHFS), a population-based cohort study of Black breast cancer survivors, both of which have been described extensively in our previous work and in the Additional file 1: Methods [14–17]. The WCHS and WCHFS used the same methods for recruitment, interviews, and eligibility. Briefly, participants were 20–75 years old; self-identified as Black or White (for WCHS); had primary, histologically confirmed invasive breast cancer or ductal carcinoma in situ (DCIS); and had no previous history of cancer other than non-melanoma skin cancer. Women in WCHS were diagnosed between 2001 and 2013 and included Black and White cases from New York City and New Jersey; while cases in WCHFS included only Black women diagnosed from 2013 to 2019 in New Jersey. Clinical and tumor pathology variables were extracted from the pathology reports. All women provided informed consent and the study protocol was approved by the Institutional Review Boards at Rutgers Cancer Institute of New Jersey and Roswell Park Comprehensive Cancer Center.

### Tissue samples

Formalin-fixed and paraffin-embedded (FFPE) invasive breast tumor tissues were built into tissue microarrays (TMAs) under the guidance of an experienced breast pathologist (TK). TMA cores ranged in size from 0.6 to 1.2 mm in diameter, and the majority of patient tumors (67.2%) were represented by at least 3 TMA cores (range 1–6 cores). We aimed to include both tumor nests and stromal regions when selecting regions for coring and avoid the tumor margins. TMA construction was completed in 2017 from patients recruited up until this point with incident, primary, and treatment-naïve invasive breast cancer. As the WCHS and WCHFS focused on recruiting Black women, the number of cases from Black women in our dataset exceeds the number of White cases (Black: N = 507, White: N = 104).

### Immunohistochemical staining and image analysis

CD163 has long been established as a clinical antibody for detecting histiocytes that has greater specificity than CD68 [18], and is commonly used to represent immunosuppressive macrophages in the TIME in research studies [19]. Immunohistochemistry (IHC) was performed by the Pathology Network Shared Resource at Roswell Park following standard procedures. To reduce staining variability that can occur with IHC, we used an automated staining platform, clinical-grade reagents, and stained all TMAs in a single batch. Briefly, TMA sections were cut at 4 μm, placed on charged slides, dried, and deparaffinized. Bond Epitope Retrieval 2 (Leica AR9640) was used for antigen retrieval. Slides were stained on the Leica Bond Rx autostainer with the CD163 antibody (Leica Biosystems, clone 10D6) and the Bond Polymer Refine Detection kit (Leica DS9800). Diaminobenzidine (DAB) was used for marker visualization. TMA cores were excluded if the tumor could not be reliably scored for CD163 marker expression (e.g., the tissue was folded or damaged) or there was insufficient tumor cellularity (cutoff set at 100 tumor cells).

Slides were digitally scanned using Aperio AT2 (Leica Biosystems, Inc., Buffalo Grove, IL) with 20X bright-field microscopy. Aperio ImageScope version 12.4.3.8007 (Leica Biosystems, Inc., Buffalo Grove, IL) was used for image analysis. Slide image data fields were populated, and images were visually examined for quality and amended as necessary (e.g., core excluded if there was excessive folding or damage). An annotation layer was created for each core and our study pathologist who was blinded to sample characteristics made an image analysis algorithm macro that was used to quantify the number of cells that were positive for CD163 stain. Details pertaining to the algorithm and scoring are described in the Additional file 1: Methods. The number of CD163^+^ cells in each patient sample were reported per square millimeter of tumor tissue and the average CD163^+^ cell density across multiple cores from each patient was used for analyses.

### Epidemiological and tumor variables

Women self-identified their race in the baseline questionnaire. Tumor and clinicopathological factors were abstracted from the patient pathology report and included AJCC stage, grade, tumor size, node status, and treatment (surgery, chemotherapy, radiation therapy, and/or hormone therapy). Breast cancer subtypes were inferred from estrogen receptor (ER), progesterone receptor (PR), and human epidermal growth factor receptor 2 (HER2) status information from the pathology reports as follows: luminal (HR+/HER2−), HER2-positive (HR+/HER2+ or HR-/HER2+), and triple-negative (HR-/HER2−), where hormone receptor (HR+) refers to ER+ and/or PR+. Other factors, including age and body mass index (BMI), were obtained by interviewer- and self-administered questionnaires at baseline and have been previously described [20].

### Breast cancer outcomes

Data on vital status, including dates and causes of death, were ascertained through linkage with the New Jersey State Cancer Registry files, and were available for 546 cases. Primary outcomes of interest in the study were overall survival (OS) and breast cancer-specific survival (BCSS). The ICD-10 code (C50) was used to identify breast cancer mortality. Time to follow-up was calculated from the date of diagnosis until the date of last follow-up (August 2023) or death from any cause or death from breast cancer.

### Statistical analyses

Demographic and clinical factors were summarized using the mean and standard deviation for normally distributed continuous variables and the median and interquartile range (IQR) otherwise, and number and percentage for categorical variables. A negative binomial regression model was used to resolve overdispersion of CD163^+^ cell density and non-normally distributed residuals seen with a linear model. A zero-inflation parameter was included due to underfitting of zero values and an offset term for the log of total cell density to account for tumor cellularity differences across patients. Model assumptions were verified graphically. Beta coefficients were exponentiated to obtain Rate Ratios (RR) and 95% Confidence Intervals (CI) representing the change in CD163^+^ cell density in terms of percentage increase or decrease. Separate models were used to model CD163^+^ cell density as a function of race and clinical/tumor factors. F tests about the appropriate contrasts of model estimates were used to evaluate, within race, the association between CD163^+^ cell density and each factor. A multivariable model was formulated to assess the association between race and CD163^+^ macrophage density, adjusted for age, subtype, grade, and tumor size.

Multivariable Cox regression models were used to compute hazard ratios (HRs) and 95% confidence intervals (CIs) for the association of CD163^+^ cell density with OS and BCSS for each breast cancer subtype. As there are currently no established cutoffs in the literature, CD163^+^ cell density was divided into tertiles. Other cutoffs were examined, including dividing CD163^+^ cell density at the median, and by quantiles and quintiles. Variables that were significantly associated with CD163^+^ cell density or survival in the univariate setting were added to a multivariable model and sequentially removed while assessing model fit using a likelihood ratio test (Additional file 1: Tables S1 and S2). Covariates were retained in the final model if their inclusion improved model fit. Model covariates differed by breast cancer subtype. Model 1 was adjusted for age at diagnosis. For OS, Model 2 was adjusted for age, BMI, stage, and tumor size in the luminal subtype; age plus tumor size for the HER2+ subtype; and age, stage, grade, and node status for the triple-negative subtype. For BCSS, Model 2 was adjusted for age and BMI in the luminal subtype; no additional covariates were retained for the HER2+ subtype; and age and stage for the triple-negative subtype. The proportional hazards assumption was verified graphically by analyzing the correlation between time and scaled Schoenfeld residuals. All statistical analyses were conducted in R (version 4.2.0) and two-sided *p* values ≤ 0.05 were considered statistically significant. Analyses are reported according to REMARK guidelines [21].

## Results

### Characteristics of the cohort

Cohort characteristics are shown in Table 1 and the study sampling schema is shown in Additional file 1: Figure S1. In total, there were 611 women with invasive breast cancer (507 Black and 104 White); of these 546 women had available survival data. Compared with White women, Black women were significantly more likely to have higher BMI (30.5 vs 26.6 kg/m^2^), have tumors that were ER-negative (33.5 vs 21.2, *p* = 0.01), triple-negative (25.5 vs 14.4, *p* = 0.04), and tumors with higher grade (54.1 grade 3 vs 35.6 *p* = 0.003). Black women were also more likely than White women to have received radiation therapy (68.5 vs 46.5, *p* < 0.001). There were no statistically significant differences between Black and White women in age, the distribution of breast cancer stage, mean tumor size, node status, and the receipt of surgery, chemotherapy, or hormone therapy.

**Table 1:** Patient descriptive characteristics

Characteristic	Overall	Black	White	*p*-value
Total	611	507 (83.0)	104 (17.0)	
Age at diagnosis, Mean (SD)	53.6 (11.0)	53.8 (11.1)	52.5 (10.6)	0.25
BMI (kg/m^2^), Median (IQR)	29.9 (26.2–34.7)	30.5 (26.8–35.6)	26.6 (22.8–30.9)	< 0.001
ER status				0.01
ER+	419 (68.6)	337 (66.5)	82 (78.8)	
ER-	192 (31.4)	170 (33.5)	22 (21.2)	
Subtype				0.04
Luminal	359 (59.2)	293 (58.4)	66 (63.5)	
HER2+	104 (17.2)	81 (16.1)	23 (22.1)	
Triple negative	143 (23.6)	128 (25.5)	15 (14.4)	
Stage				0.96
I	260 (42.6)	217 (42.8)	43 (41.3)	
II	269 (44.0)	222 (43.8)	47 (45.2)	
III/IV	82 (13.4)	68 (13.4)	14 (13.5)	
Grade				0.003
1	82 (13.6)	63 (12.6)	19 (18.8)	
2	213 (35.4)	167 (33.3)	46 (45.5)	
3	307 (51.0)	271 (54.1)	36 (35.6)	
Tumor size (cm), Median (IQR)	2.0 (1.3–2.8)	2.0 (1.3–3.0)	1.9 (1.2–2.5)	0.28
Node status				0.45
Positive	242 (40.4)	197 (39.7)	45 (43.7)	
Negative	357 (59.6)	299 (60.3)	58 (56.3)	
Surgery				0.71
No	13 (2.2)	12 (2.4)	1 (1.0)	
Yes	591 (97.8)	489 (97.6)	102 (99.0)	
Chemotherapy				0.35
No	200 (33.1)	170 (33.9)	30 (29.1)	
Yes	404 (66.9)	331 (66.1)	73 (70.9)	
Radiation				< 0.001
No	212 (35.2)	158 (31.5)	54 (53.5)	
Yes	390 (64.8)	343 (68.5)	47 (46.5)	
Hormone therapy				0.75
No	226 (37.5)	186 (37.2)	40 (38.8)	
Yes	377 (62.5)	314 (62.8)	63 (61.2)	
Vital status				
Alive	419 (76.7)	361 (77.5)	58 (72.5)	
Breast cancer-specific death	66 (12.1)	54 (11.6)	12 (15.0)	
Non-breast cancer-specific death	61 (11.2)	51 (10.9)	10 (12.5)	
Follow-up time (years), median (IQR)	9.7 (7.4–12.3)	9.3 (7.2–11.0)	15.1 (13.4–16.1)	

Normally distributed continuous variables presented as mean (SD) and non-normally distributed variables shown as median (IQR). Categorical variables are shown as N (%). Differences between self-identified race assessed using Welch Two Sample t-test for normally distributed continuous variables, Wilcoxon rank sum test for non-normally distributed continuous variables, and Pearson’s Chi-squared or Fisher’s exact test for categorical variables

Missingness as follows: BMI (1), subtype (5), grade (9), tumor size (5), node status (12), surgery (7), chemotherapy (7), radiation (9), hormone therapy (8), vital status (65), follow-up time (65)

*BMI* body mass index (calculated as weight in kilograms divided by height in meters squared), *ER* Estrogen receptor

### Macrophage densities, race, and clinical prognostic factors

**Fig. 1:**
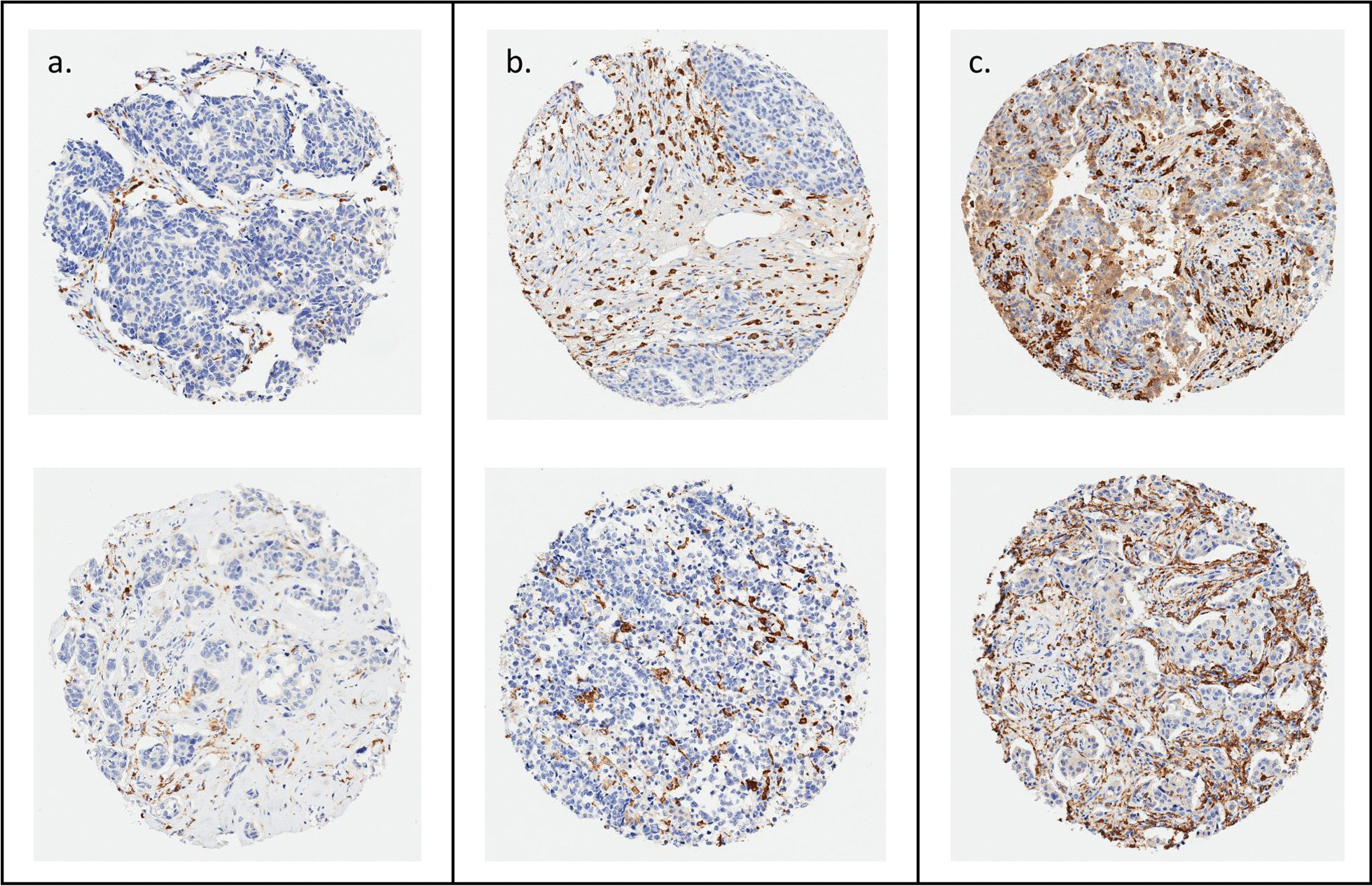
Representative CD163 immunohistochemical staining in breast tissue microarray cores. Two representative cores are shown from each of three categories of infiltration: **a** low, **b** intermediate, **c** high

**Fig. 2:**
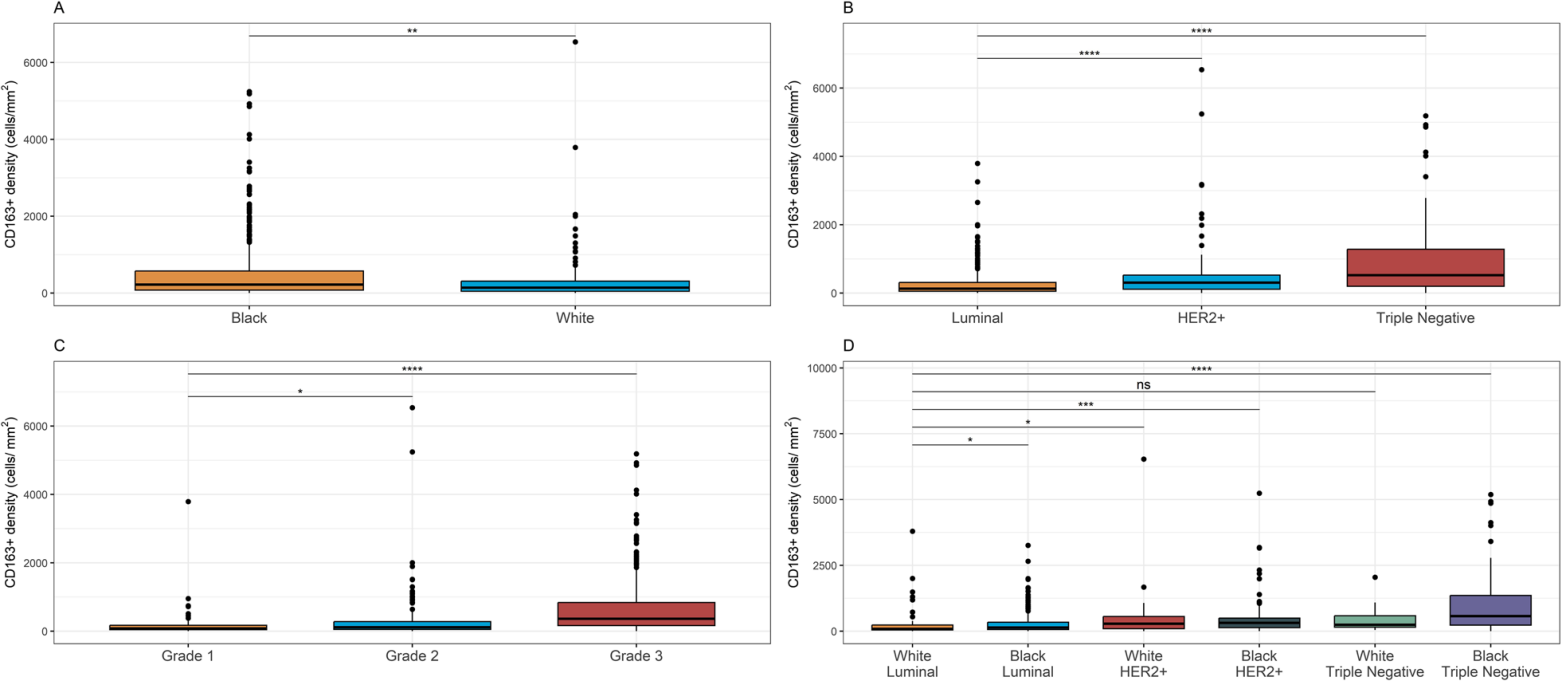
Boxplots comparing CD163^+^ cell density by **a** race, **b** breast cancer subtype, **c** tumor grade, and **d** combination of race and breast cancer subtype. Comparisons tested using negative binomial regression. *ns* non-significant, *p < 0.05, **p < 0.01, ***p < 0.001, ****p < 0.0001

Staining is shown for cores representative of low, intermediate, and high levels of CD163^+^ macrophage infiltration in Fig. 1. Almost all women in the WCHS had macrophages in their tumors; CD163^+^ macrophages were not detected in only 6 out of 611 women. In univariate analyses, Black women had a significantly higher density of CD163^+^ cells (*p* = 0.0099, Fig. 2a). CD163^+^ macrophage densities were also higher in triple-negative tumors (*p* < 0.0001, Fig. 2b), and higher-grade tumors (*p* < 0.0001, Fig. 2c). Black women with the triple-negative subtype (median 574.3 cells/μm^2^, *p* < 0.001), Black women with the HER2 + subtype (314.6 cells/μm^2^, *p* < 0.001), and White women with the HER2 + subtype (281.5 cells/μm^2^, *p* = 0.035) had significantly higher densities of CD163^+^ macrophages compared to White women with the luminal subtype (Fig. 2d). In the overall study population, CD163^+^ macrophage density was significantly associated with age (*p* = 0.025), breast cancer subtype (*p* < 0.001), stage (*p* < 0.001), grade (*p* < 0.001), and tumor size (*p* = 0.002); similar associations were observed when the Black population was examined separately (Table 2). In a multivariate negative binomial regression model that adjusted for age, subtype, grade, and tumor size, there were no significant differences in CD163^+^ macrophage densities between Black and White women (RR = 0.88; 95% CI 0.71–1.10). To investigate a possible cohort effect given that recruitment for White women ended earlier than that for Black women, we compared Black and White cases up until the last timepoint that White women were enrolled and observed similar results (RR = 0.88; 95% CI 0.67–1.16).

**Table 2:** Univariate negative binomial regression models assessing associations between cohort characteristics with CD163^+^ cell density in the overall sample and within Black and White cases

Characteristic	Overall			Black			White		
	n	Median (IQR)^a^	*p*-value	n	Median (IQR)^a^	*p*-value	n	Median (IQR)^a^	*p*-value
Total	611	203.7 (451.3)		507	223.2 (497.2)		104	142.5 (266.6)	
Age at diagnosis			0.025			0.054			0.083
< 40	63	314.6 (836.4)		52	347.4 (924.7)		11	141.5 (540.3)	
40–50	158	191.5 (369.6)		125	207.3 (417.8)		33	156.4 (251.5)	
50–60	201	197.0 (453.8)		169	201.4 (502.1)		32	118.6 (268.5)	
60+	189	207.3 (371.5)		161	225.4 (402.0)		28	143.9 (273.7)	
BMI			0.81			0.32			0.47
< 25	123	191.5 (430.5)		81	246.2 (753.9)		42	118.3 (234.5)	
25–30	184	174.2 (431.3)		153	188.8 (421.1)		31	141.5 (410.2)	
30–35	154	243.4 (526.4)		137	255.0 (525.5)		17	104.1 (238.8)	
35+	149	221.6 (423.4)		135	221.4 (488.7)		14	276.1 (178.8)	
ER status			< 0.001			< 0.001			0.10
ER+	419	140.3 (295.2)		337	153.1 (303.3)		82	99.5 (229.1)	
ER−	192	436.3 (884.4)		170	466.8 (961.0)		22	296.8 (623.7)	
Subtype			< 0.001			< 0.001			0.14
Luminal	359	130.8 (256.8)		293	139.0 (278.6)		66	88.7 (194.0)	
HER2+	104	308.3 (413.8)		81	314.6 (364.9)		23	281.5 (466.5)	
Triple negative	143	526.3 (1,083.1)		128	574.3 (1,127.0)		15	245.0 (431.5)	
Stage			< 0.001			0.002			0.20
I	260	149.6 (310.0)		217	187.7 (343.4)		43	84.5 (161.1)	
II	269	243.0 (521.0)		222	258.3 (628.7)		47	167.1 (288.4)	
III/IV	82	237.9 (484.8)		68	242.2 (427.9)		14	183.9 (593.4)	
Grade			< 0.001			< 0.001			0.022
1	82	82.0 (130.5)		63	84.0 (156.1)		19	52.5 (123.5)	
2	213	114.2 (226.0)		167	130.5 (247.9)		46	79.8 (129.7)	
3	307	362.5 (678.6)		271	365.3 (689.2)		36	321.1 (591.9)	
Tumor size (cm)			0.002			0.005			0.48
< 1	67	111.9 (280.9)		55	130.5 (309.2)		12	33.9 (106.0)	
1–2	233	192.1 (363.5)		190	207.3 (430.6)		43	143.5 (234.3)	
2+	306	236.7 (521.5)		257	261.7 (535.3)		49	165.6 (300.8)	
Node status			0.44			0.59			0.33
Positive	242	211.9 (463.2)		197	221.6 (494.1)		45	165.6 (321.1)	
Negative	357	199.6 (449.4)		299	227.9 (493.7)		58	108.5 (237.9)	

*BMI* body mass index, *ER* Estrogen receptor status

^a^CD163^+^ Cell Density Median (IQR)

### Survival outcomes and CD163^+^ macrophages

Data for survival analyses were available for 546 women, with 127 deaths, 66 of which were due to breast cancer. The median follow-up time was 9.68 years (IQR: 7.43–12.33) years. For the overall cohort, increasing tertiles of CD163^+^ macrophage density were not associated with a significant improvement in OS or BCSS in the age-adjusted models (Table 3). For the fully adjusted models, there was a significant association for OS (Q3 vs. Q1: HR = 0.59; 95% CI 0.37–0.94), but not BCSS (Q3 vs. Q1: HR = 0.59; 95% CI 0.30–1.14; Table 3). In both age-adjusted and fully adjusted models stratified by subtype, increasing tertiles of CD163^+^ macrophage density were associated with a significant improvement in OS (Q3 vs. Q1: HR = 0.30; 95% CI 0.12–0.73; Table 4) in the triple-negative subtype. A statistically significant association between CD163^+^ macrophage densities and OS was not observed for the luminal and HER2+ subtypes. A similar pattern was observed for BCSS, in which increasing CD163^+^ macrophage densities were associated with better survival in the triple-negative subtype only (Q3 vs. Q1: HR = 0.38; 95% CI 0.10–1.44), although the associations were not significant (Table 4).

**Table 3:** Multivariable Cox regression models assessing associations of CD163^+^ cell density tertiles with overall survival and breast cancer-specific survival in the overall cohort

Model	Characteristic	Overall survival			Breast cancer-specific survival		
		Events/N	HR (95% CI)^1^	*p*-value	Events/N	HR (95% CI)^1^	*p*-value
Model 1	CD163						
	T1	47/182	–		24/182	–	
	T2	41/182	0.92 (0.61–1.40)	0.71	20/182	0.88 (0.49–1.60)	0.69
	T3	39/182	0.95 (0.62–1.45)	0.81	17/182	0.82 (0.44–1.52)	0.52
	Age	127/546	1.18 (1.09–1.28)	< 0.001	61/546	1.48 (1.29–1.69)	< 0.001
Model 2	CD163						
	T1	45/180	–		24/181	–	
	T2	37/177	0.64 (0.41–1.01)	0.056	19/180	0.74 (0.40–1.38)	0.34
	T3	38/180	0.59 (0.37–0.94)	0.026	17/181	0.59 (0.30–1.14)	0.11
	Age	120/537	1.27 (1.16–1.39)	< 0.001	60/542	1.52 (1.33–1.75)	< 0.001
	Stage						
	I	36/236	–		25/236	–	
	II	47/240	1.04 (0.65–1.68)	0.86	25/242	1.21 (0.69–2.14)	0.51
	III/IV	37/61	4.77 (2.65–8.59)	< 0.001	10/64	3.21 (1.51–6.79)	0.002
	Subtype						
	Luminal	62/327	–		34/330	–	
	Triple negative	39/119	2.56 (1.64–3.98)	< 0.001	17/120	2.55 (1.38–4.71)	0.003
	HER2+	19/91	1.83 (1.06–3.15)	0.030	9/92	1.29 (0.61–2.74)	0.51
	Tumor size	120/537	1.18 (1.06–1.30)	0.002			
	BMI	120/537	1.20 (1.05–1.36)	0.006			

CD163^+^ cell density categorized into tertiles based on the overall cohort distribution

Model 1 adjusted for age at diagnosis. OS/Model 2 adjusted for age, stage, subtype, tumor size, and BMI. BCSS/Model 2 adjusted for age, stage, and subtype

Age, BMI, and tumor size modeled continuously reflecting a 5-year increase in age, a 5-kg/m^2^ increase in BMI, and 1 cm increase in tumor size

^1^*HR* Hazard Ratio, *CI* Confidence Interval

**Table 4:** Multivariable Cox regression models assessing associations between CD163^+^ cell density tertiles with overall survival and breast cancer-specific survival within subtype

Subtype, model		Overall survival			Breast cancer-specific survival		
Luminal	Characteristic	Events/N	HR (95% CI)^1^	*p*-value	Events/N	HR (95% CI)^1^	*p*-value
Model 1	CD163						
	T1	24/110	–		10/110	–	
	T2	17/110	0.81 (0.43–1.51)	0.50	8/110	0.99 (0.39–2.52)	0.98
	T3	24/110	0.99 (0.56–1.76)	0.98	16/110	1.40 (0.63–3.13)	0.41
	Age	65/330	1.25 (1.10–1.42)	< 0.001	34/330	1.56 (1.26–1.92)	< 0.001
Model 2	CD163						
	T1	23/109	–		10/110	–	
	T2	15/108	0.54 (0.28–1.07)	0.076	8/109	0.99 (0.39–2.52)	0.98
	T3	24/110	0.83 (0.46–1.52)	0.55	16/110	1.27 (0.57–2.86)	0.56
	Age	62/327	1.28 (1.11–1.47)	< 0.001	34/329	1.55 (1.25–1.93)	< 0.001
	BMI	62/327	1.27 (1.07–1.51)	0.006	34/329	1.29 (1.03–1.61)	0.027
	Stage						
	I	26/166	–				
	II	17/127	0.58 (0.30–1.16)	0.12			
	III/IV	19/34	3.44 (1.69–6.99)	< 0.001			
	Tumor size (cm)	62/327	1.24 (1.08–1.42)	0.002			

Subtype, model		Overall survival			Breast cancer-specific survival		
HER2+	Characteristic	Events/N	HR (95% CI)^1^	*p*-value	Events/N	HR (95% CI)^1^	*p*-value
Model 1	CD163						
	T1	6/31	–		4/31	–	
	T2	6/31	0.99 (0.31–3.15)	0.99	2/31	0.42 (0.07–2.40)	0.33
	T3	8/30	1.37 (0.47–4.04)	0.57	3/30	0.54 (0.11–2.64)	0.45
	Age	20/92	1.26 (1.03–1.54)	0.024	9/92	1.78 (1.21–2.61)	0.003
Model 2	CD163						
	T1	6/31	–				
	T2	6/31	0.59 (0.16–2.16)	0.43			
	T3	7/29	0.67 (0.19–2.31)	0.52			
	Age	19/91	1.34 (1.09–1.66)	0.007			
	Tumor size (cm)	19/91	1.70 (1.11–2.60)	0.015			

Subtype, model		Overall survival			Breast cancer-specific survival		
Triple negative	Characteristic	Events/N	HR (95% CI)^1^	*p*-value	Events/N	HR (95% CI)^1^	*p*-value
Model 1	CD163						
	T1	20/40	–		9/40	–	
	T2	12/40	0.59 (0.29–1.21)	0.15	5/40	0.61 (0.20–1.85)	0.38
	T3	8/40	0.36 (0.16–0.83)	0.017	3/40	0.36 (0.10–1.38)	0.14
	Age	40/120	1.07 (0.93–1.23)	0.34	17/120	1.26 (1.01–1.58)	0.041
Model 2	CD163+						
	T1	18/38	–		9/40	–	
	T2	11/38	0.44 (0.19–1.01)	0.052	5/40	0.70 (0.23–2.13)	0.53
	T3	8/40	0.30 (0.12–0.73)	0.008	3/40	0.38 (0.10–1.44)	0.15
	Age	37/116	1.28 (1.07–1.53)	0.008	17/120	1.38 (1.08–1.78)	0.012
	Stage						
	I	5/34	–		3/35	–	
	II	17/60	1.46 (0.46–4.64)	0.52	11/62	3.09 (0.82–11.7)	0.10
	III/IV	15/22	7.60 (2.01–28.7)	0.003	3/23	6.66 (1.23–36.2)	0.028
	Grade						
	3	32/99	–				
	1/2	5/17	0.33 (0.11–1.01)	0.053			
	Node status						
	Positive	23/47	–				
	Negative	14/69	0.47 (0.20–1.13)	0.093			

CD163^+^ cell density categorized into tertiles based on the distribution within subtype, separately

Model 1 adjusted for age across all subtypes and outcomes. Model 2 adjusted for parsimonious set of covariates found using covariate selection methods within subtype and outcome (Luminal/OS: age, BMI, stage, tumor size; Luminal/BCSS: age, BMI. HER2+ /OS: age, tumor size; HER2+ /BCSS: age. Triple Negative/OS: age, stage, grade, node status; Triple Negative/BCSS: age, stage)

Age, BMI, and tumor size modeled continuously reflecting a 5-year increase in age, a 5-kg/m^2^ increase in BMI, and 1 cm increase in tumor size

^1^*HR* Hazard Ratio, *CI* Confidence Interval

To ensure that race and grade were not confounding the associations that we observed in the triple-negative subtype, additional multivariable analyses that added race and grade as variables in the fully adjusted models were investigated. Again, we observed that increasing CD163^+^ macrophage density was associated with a significant improvement in OS for the triple-negative subtype (Q3 vs. Q1: HR = 0.28; 95% CI 0.11–0.69), but not for the luminal or HER2+ subtypes (Additional file 1: Table S3). Several additional sensitivity analyses were performed to ensure our results were robust. Additional cut points of CD163 marker density were examined, such as dividing at the cohort median to differentiate high vs low CD163 density, as well as quantiles and quintiles (Additional file 1: Tables S4 and S5). We stratified by ER status rather than breast cancer subtype (Additional file 1: Table S6). Lastly, we performed the analysis in Black patients only (Additional file 1: Table S7). For all these additional analyses, we observed that increasing levels of CD163^+^ macrophage infiltration were associated with improved OS in the triple-negative subtype (or ER-negative group for analyses stratified by ER status), and this effect was not observed for the luminal or HER2 + subtypes.

## Discussion

In this study, we found that increasing densities of CD163^+^ macrophages in the breast TIME were associated with a pronounced and significant improvement in OS for women with the triple-negative subtype. Prior studies investigating the association between TAMs and breast cancer prognosis have contributed to a general consensus that high levels of TAMs in the breast TIME, especially M2-like macrophages, are associated with adverse survival outcomes [5–7, 9, 22]. So, what might explain the differing results in our study? First, we have a relatively large population of Black women allowing us to stratify by subtype and adjust for confounding factors. As subtypes of breast cancer differ in their patterns of short and long-term survival, stratification by subtype can reveal different associations in relation to prognostic or risk factors [23–25]. This holds true for patterns of immune infiltration in the breast TIME that are known to vary by subtype and show differing associations with survival [1, 26, 27]. The majority of prior studies that examined TAM infiltration in breast carcinoma were underpowered for subtype-specific associations, especially for the triple-negative subtype, in which sample sizes were extremely small [5, 6, 9, 11].

Second, macrophages are a complex immune cell population with a variety of phenotypes and functional states that can be tissue specific and dependent on microenvironmental cues and/or spatial proximity to other immune subsets [28–30]. Moreover, there are no standardized methods for macrophage detection and different studies have used different markers (e.g., CD68, CD163, or CD206) and staining platforms to make conclusions about the prognostic value of macrophages in invasive breast cancer. Methods for quantifying macrophages in the breast TIME are also heterogeneous (e.g., density, percentage) as well as the tissue compartment in which macrophages are assessed (e.g., tumor compartment vs. stroma or both). The cutoff values for what constitute high versus low macrophage infiltration also varies by study, as well as what factors are included in multivariable models.

We conducted several quality controls and performed several sensitivity analyses to ensure that our findings were robust. First, we used a clinical-grade CD163 antibody that is approved for in vitro diagnostic purposes. Second, quality control for staining specificity was performed by an experienced breast pathologist. Third, automated image analysis was performed ensuring that the quantification of CD163 positive cells was standardized and objective across each TMA core. Fourth, all TMAs were stained in a single batch to eliminate inter-batch variability that is known to occur with IHC. From an analysis standpoint, we examined different cutoffs for what constitutes high or low CD163^+^ macrophage infiltration, dividing the cohort at the median, tertiles, quantiles, and quintiles. We examined associations when stratifying by ER status instead of subtype. Lastly, we examined Black women separately. The same general patterns of improved OS and BCSS in the triple-negative subtype (or ER- group) were observed across all these additional analyses.

As shown in our results and in the literature, high macrophage infiltration in breast cancer is correlated with several factors that indicate poor survival, like the triple-negative subtype, and higher grade and stage [5–8]. In prior studies that could not account for these factors, the associations of high macrophage densities with poor survival may have been largely driven by these correlated factors. A recent study that investigated multiple macrophage markers in relation to breast cancer outcomes showed that when examining the ER-positive versus ER-negative groups separately, high expression of CD163 was associated with improved OS in ER− cases, but not in ER+ cancers [31]. When examining CD163 expression by tumor locations, Fortis et al. found that disease-free survival (DFS) and OS were prolonged in patients with CD163 expression that was low in the tumor center but high at the invasive margins compared to the inverse (i.e., high in tumor center and low in the invasive margin) [32]. Collectively, these findings together with those reported in our study add to the existing body of evidence suggesting that tumor-associated macrophages have distinct programs that vary by tissue context or breast cancer subtype. While CD163^+^ macrophages are usually regarded as immune-suppressing and tumor-promoting, human macrophages are likely to concurrently exhibit phenotypic characteristics of both M1-like and M2-like subtypes. Therefore, to gain a broader appreciation of the macrophage response in breast cancer outcomes, phenotypic studies combined with comprehensive functional and transcriptomic analyses may strengthen translational relevance to prognosis.

Univariate analyses showed that CD163^+^ cell densities differed between Black and White women, but these differences were attenuated in the multivariable analyses that adjusted for age, grade, tumor size, and breast cancer subtype. Earlier work has shown that immune profiles vary in breast tumors from Black and White women [14, 15, 33, 34]. While other studies have compared macrophage markers in Black and White women, to our knowledge, only a couple studies have compared CD163 marker expression specifically [9, 10]. Koru-Sengul et al. reported that Black women had higher levels of CD163^+^ macrophages, however multivariable analyses were not performed [11]. In a more recent study, Bauer et al. found that the frequency of CD163^+^ macrophages varied by region within African populations and a population from Germany; West African women had the highest numbers of CD163^+^ macrophages [35].

The strengths of this work are accompanied by some limitations. While our study sample exceeds that of several prior studies of CD163 in relation to breast cancer prognosis, it is nonetheless not as large as some of the more well-characterized T cell populations like CD8^+^ T cells [4], and our findings need to be replicated in additional cohorts. As the WCHS and WCHFS prioritized recruitment of Black women, our findings may not be generalizable to more demographically or clinically diverse populations. As the vast majority (89.5%) of our cases were obtained through the New Jersey Cancer registry, our sample is largely population-based. Nonetheless, potential sources of bias include women who agreed to participate verses those who did not. However, the distributions of tumor stage and grade are similar among participants in the WCHFS and all eligible breast cancer cases in the New Jersey State Cancer Registry in the same counties, suggesting that tumor characteristics in our study are representative of Black women diagnosed with breast cancer in New Jersey [16]. Recall bias is minimized as the data pertaining to the tumor characteristics were obtained by independent review of pathology reports. Despite adjusting for important clinical and demographic prognostic factors, we cannot rule out the possibility of residual confounding due to unmeasured variables. Lastly, although whole sections are ideal for studies of the TIME, a study of this size is not practicable with whole sections, and therefore TMAs are commonly used in large studies of marker expression in breast cancer [4, 36, 37]. Importantly, we cored the interior of the tumor block for TMA construction and thus our results are specific to this region and do not apply to the tumor interface or other non-tumor regions. Macrophages are a complex population and our future work will build on this fundamental finding, making use of multiplexed panels to more fully define macrophage phenotypes in women with invasive breast cancer, as well as their spatial distribution, which could further influence their prognostic relevance [32].

## Conclusion

We observed that higher densities CD163^+^ macrophages are independently associated with improved OS and BCSS in the triple-negative subtype. Future investigations will expand upon this work in a larger cohort, incorporating more comprehensive multiplexed staining technologies to further define the complexity of macrophage functional states and compare their localization within the TIME to prognosis in women with invasive breast cancer.

### Supplementary Information

**Additional file 1: Table S1.** Univariate Cox regression models assessing associations of additional CD163^+^ cell density cutoffs and cohort characteristics with overall survival (OS) within subtype. **Table S2.** Univariate Cox regression models assessing associations of additional CD163^+^ cell density cutoffs and cohort characteristics with breast cancer-specific survival (BCSS) within subtype. **Table S3.** Multivariable Cox regression models assessing associations between CD163^+^ cell density tertiles with OS and BCSS within subtype, additionally adjusting for self-identified race and grade in Model 2. **Table S4.** Multivariable Cox regression models assessing associations of additional CD163^+^ cell density cutoffs with OS within subtype. **Table S5.**. Multivariable Cox regression models assessing associations of additional CD163^+^ cell density cutoffs with BCSS within subtype. **Table S6.** Multivariable Cox regression models assessing associations between CD163^+^ cell density tertiles with OS and BCSS by estrogen receptor (ER) status. **Table S7.** Multivariable Cox regression models assessing associations between CD163^+^ cell density tertiles with OS and BCSS within Black cases. **Figure S1.** Diagram of participant availability for CD163 profiling in the Women’s Circle of Health Study.

## Data Availability

Epidemiological data and image data are available from the corresponding author upon reasonable request.
