# Supplementary material for: CD163+ macrophages in the triple-negative breast tumor microenvironment are associated with improved survival in the Women’s Circle of Health Study and the Women’s Circle of Health Follow-Up Study
Source: Breast Cancer Res. 2024 May 8;26:75. doi: 10.1186/s13058-024-01831-8 (PMC11077737; doi:10.1186/s13058-024-01831-8)
Supplement: Supplementary file 1 — Additional file 1: Table S1. Univariate Cox regression models assessing associations of additional CD163+ cell density cutoffs and cohort characteristics with overall survival (OS) within subtype. Table S2. Univariate Cox regression models assessing associations of additional CD163+ cell density cutoffs and cohort characteristics with breast cancer-specific survival (BCSS) within subtype. Table S3. Multivariable Cox regression models assessing associations between CD163+ cell density tertiles with OS and BCSS within subtype, additionally adjusting for self-identified race and grade in Model 2. Table S4. Multivariable Cox regression models assessing associations of additional CD163+ cell density cutoffs with OS within subtype. Table S5.. Multivariable Cox regression models assessing associations of additional CD163+ cell density cutoffs with BCSS within subtype. Table S6. Multivariable Cox regression models assessing associations between CD163+ cell density tertiles with OS and BCSS by estrogen receptor (ER) status. Table S7. Multivariable Cox regression models assessing associations between CD163+ cell density tertiles with OS and BCSS within Black cases. Figure S1. Diagram of participant availability for CD163 profiling in the Women’s Circle of Health Study. [file 13058_2024_1831_MOESM1_ESM.docx]

Supplemental Tables, Figures, and Methods file

Supplemental Tables

Table S1. Univariate Cox regression models assessing associations of additional CD163^+^ cell density cutoffs and cohort characteristics with overall survival (OS) within subtype

Table S2. Univariate Cox regression models assessing associations of additional CD163^+^ cell density cutoffs and cohort characteristics with breast cancer-specific survival (BCSS) within subtype

Table S3. Multivariable Cox regression models assessing associations between CD163^+^ cell density tertiles with OS and BCSS within subtype, additionally adjusting for self-identified race and grade in Model 2

Table S4. Multivariable Cox regression models assessing associations of additional CD163^+^ cell density cutoffs with OS within subtype

Table S5. Multivariable Cox regression models assessing associations of additional CD163^+^ cell density cutoffs with BCSS within subtype

Table S6. Multivariable Cox regression models assessing associations between CD163^+^ cell density tertiles with OS and BCSS by estrogen receptor (ER) status

Table S7. Multivariable Cox regression models assessing associations between CD163^+^ cell density tertiles with OS and BCSS within Black cases

Supplemental Figures

Figure S1. Diagram of participant availability for CD163 profiling in the Women’s Circle of Health Study

Table S1. Univariate Cox regression models assessing associations of additional CD163^+^ cell density cutoffs and cohort characteristics with overall survival within subtype

|  | Luminal | | | HER2+ | | | Triple Negative | | |
| --- | --- | --- | --- | --- | --- | --- | --- | --- | --- |
| Characteristic | Events/N | HR (95% CI)^1^ | p-value | Events/N | HR (95% CI)^1^ | p-value | Events/N | HR (95% CI)^1^ | p-value |
| CD163+ Median |  |  |  |  |  |  |  |  |  |
| Q1 | 37/165 | — |  | 7/46 | — |  | 27/60 | — |  |
| Q2 | 28/165 | 0.74 (0.45 to 1.21) | 0.23 | 13/46 | 2.33 (0.92 to 5.85) | 0.073 | 13/60 | 0.40 (0.21 to 0.78) | 0.007 |
| CD163+ Tertile |  |  |  |  |  |  |  |  |  |
| Q1 | 24/110 | — |  | 6/31 | — |  | 20/40 | — |  |
| Q2 | 17/110 | 0.75 (0.40 to 1.39) | 0.36 | 6/31 | 1.25 (0.40 to 3.89) | 0.70 | 12/40 | 0.57 (0.28 to 1.17) | 0.13 |
| Q3 | 24/110 | 1.09 (0.62 to 1.92) | 0.77 | 8/30 | 1.62 (0.56 to 4.68) | 0.37 | 8/40 | 0.33 (0.15 to 0.75) | 0.008 |
| CD163+ Quartile |  |  |  |  |  |  |  |  |  |
| Q1 | 18/83 | — |  | 5/23 | — |  | 17/30 | — |  |
| Q2 | 19/83 | 1.34 (0.70 to 2.57) | 0.37 | 2/23 | 0.41 (0.08 to 2.14) | 0.29 | 10/30 | 0.59 (0.27 to 1.29) | 0.18 |
| Q3 | 13/82 | 0.77 (0.38 to 1.57) | 0.47 | 7/23 | 2.11 (0.66 to 6.71) | 0.21 | 6/30 | 0.30 (0.12 to 0.76) | 0.011 |
| Q4 | 15/82 | 0.98 (0.49 to 1.95) | 0.96 | 6/23 | 1.33 (0.41 to 4.38) | 0.63 | 7/30 | 0.34 (0.14 to 0.81) | 0.015 |
| CD163+ Quintile |  |  |  |  |  |  |  |  |  |
| Q1 | 13/66 | — |  | 5/19 | — |  | 14/24 | — |  |
| Q2 | 16/66 | 1.53 (0.73 to 3.19) | 0.26 | 1/19 | 0.21 (0.02 to 1.80) | 0.15 | 9/24 | 0.77 (0.33 to 1.78) | 0.54 |
| Q3 | 11/66 | 0.94 (0.42 to 2.10) | 0.88 | 5/18 | 1.23 (0.36 to 4.25) | 0.74 | 6/24 | 0.42 (0.16 to 1.09) | 0.075 |
| Q4 | 12/66 | 1.06 (0.48 to 2.34) | 0.88 | 4/18 | 1.14 (0.30 to 4.30) | 0.84 | 6/24 | 0.42 (0.16 to 1.10) | 0.078 |
| Q5 | 13/66 | 1.20 (0.56 to 2.60) | 0.64 | 5/18 | 1.24 (0.36 to 4.28) | 0.74 | 5/24 | 0.31 (0.11 to 0.85) | 0.023 |
| Race |  |  |  |  |  |  |  |  |  |
| Black | 50/279 | — |  | 17/74 | — |  | 36/109 | — |  |
| White | 15/51 | 1.14 (0.62 to 2.07) | 0.67 | 3/18 | 0.36 (0.10 to 1.31) | 0.12 | 4/11 | 0.87 (0.31 to 2.46) | 0.79 |
| Age | 65/330 | 1.26 (1.11 to 1.43) | <0.001 | 20/92 | 1.27 (1.04 to 1.55) | 0.019 | 40/120 | 1.11 (0.97 to 1.27) | 0.12 |
| BMI | 64/329 | 1.27 (1.08 to 1.49) | 0.004 | 20/92 | 1.23 (0.82 to 1.84) | 0.31 | 40/120 | 0.99 (0.79 to 1.24) | 0.92 |
| Stage |  |  |  |  |  |  |  |  |  |
| I | 26/166 | — |  | 4/35 | — |  | 6/35 | — |  |
| II | 18/128 | 0.85 (0.46 to 1.54) | 0.59 | 13/52 | 2.32 (0.76 to 7.11) | 0.14 | 18/62 | 1.64 (0.65 to 4.14) | 0.29 |
| III/IV | 21/36 | 4.90 (2.76 to 8.72) | <0.001 | 3/5 | 9.02 (1.99 to 40.8) | 0.004 | 16/23 | 7.79 (3.02 to 20.0) | <0.001 |
| Grade |  |  |  |  |  |  |  |  |  |
| 3 | 28/110 | — |  | 12/58 | — |  | 35/103 | — |  |
| 1 | 10/68 | 0.54 (0.26 to 1.12) | 0.10 | 1/6 | 0.88 (0.11 to 6.76) | 0.90 | 1/1 | 4.10 (0.55 to 30.4) | 0.17 |
| 2 | 25/147 | 0.66 (0.38 to 1.13) | 0.13 | 6/27 | 1.11 (0.42 to 2.95) | 0.84 | 4/16 | 0.61 (0.22 to 1.73) | 0.36 |
| Tumor size | 63/328 | 1.27 (1.14 to 1.40) | <0.001 | 19/91 | 1.40 (0.98 to 1.98) | 0.061 | 39/119 | 1.31 (1.15 to 1.49) | <0.001 |
| Node status |  |  |  |  |  |  |  |  |  |
| Positive | 26/118 | — |  | 11/44 | — |  | 23/47 | — |  |
| Negative | 39/210 | 0.83 (0.50 to 1.36) | 0.46 | 8/47 | 0.58 (0.23 to 1.44) | 0.24 | 14/69 | 0.31 (0.16 to 0.60) | <0.001 |
| Surgery |  |  |  |  |  |  |  |  |  |
| No | 1/1 | — |  | 1/1 | — |  | 0/0 | — |  |
| Yes | 64/329 | 0.10 (0.01 to 0.73) | 0.023 | 19/91 | 0.12 (0.02 to 0.94) | 0.043 | 40/120 |  |  |
| Chemotherapy |  |  |  |  |  |  |  |  |  |
| No | 30/158 | — |  | 1/13 | — |  | 3/8 | — |  |
| Yes | 35/172 | 1.00 (0.62 to 1.63) | >0.99 | 19/79 | 2.81 (0.38 to 21.1) | 0.31 | 37/112 | 0.88 (0.27 to 2.85) | 0.83 |
| Radiation |  |  |  |  |  |  |  |  |  |
| No | 21/102 | — |  | 9/39 | — |  | 9/29 | — |  |
| Yes | 44/228 | 0.97 (0.58 to 1.64) | 0.92 | 11/53 | 1.01 (0.42 to 2.43) | 0.99 | 31/91 | 1.19 (0.57 to 2.51) | 0.64 |
| Hormone therapy |  |  |  |  |  |  |  |  |  |
| No | 12/36 | — |  | 11/39 | — |  | 35/103 | — |  |
| Yes | 53/294 | 0.59 (0.32 to 1.11) | 0.10 | 9/53 | 0.53 (0.22 to 1.28) | 0.16 | 4/16 | 0.80 (0.28 to 2.26) | 0.68 |
| ^1^HR = Hazard Ratio, CI = Confidence Interval | | | | | | | | | |
| Age, BMI, and tumor size modeled continuously reflecting a 5-year increase in age, a 5-kg/m2 increase in BMI, and 1 centimeter increase in tumor size | | | | | | | | | |

Table S2. Univariate Cox regression models assessing associations of additional CD163^+^ cell density cutoffs and cohort characteristics with breast cancer-specific survival within subtype

|  | Luminal | | | HER2+ | | | Triple Negative | | |
| --- | --- | --- | --- | --- | --- | --- | --- | --- | --- |
| Characteristic | Events/N | HR (95% CI)^1^ | p-value | Events/N | HR (95% CI)^1^ | p-value | Events/N | HR (95% CI)^1^ | p-value |
| CD163+ Median |  |  |  |  |  |  |  |  |  |
| Q1 | 16/165 | — |  | 5/46 | — |  | 12/60 | — |  |
| Q2 | 18/165 | 1.10 (0.56 to 2.15) | 0.79 | 4/46 | 1.05 (0.28 to 3.94) | 0.94 | 5/60 | 0.34 (0.12 to 0.96) | 0.042 |
| CD163+ Tertile |  |  |  |  |  |  |  |  |  |
| Q1 | 10/110 | — |  | 4/31 | — |  | 9/40 | — |  |
| Q2 | 8/110 | 0.85 (0.34 to 2.16) | 0.73 | 2/31 | 0.70 (0.13 to 3.81) | 0.68 | 5/40 | 0.55 (0.18 to 1.66) | 0.29 |
| Q3 | 16/110 | 1.78 (0.80 to 3.92) | 0.15 | 3/30 | 0.94 (0.21 to 4.22) | 0.93 | 3/40 | 0.28 (0.07 to 1.02) | 0.054 |
| CD163+ Quartile |  |  |  |  |  |  |  |  |  |
| Q1 | 8/83 | — |  | 3/23 | — |  | 8/30 | — |  |
| Q2 | 8/83 | 1.34 (0.50 to 3.59) | 0.56 | 2/23 | 0.70 (0.12 to 4.18) | 0.69 | 4/30 | 0.49 (0.15 to 1.64) | 0.25 |
| Q3 | 9/82 | 1.21 (0.47 to 3.14) | 0.69 | 1/23 | 0.58 (0.06 to 5.63) | 0.64 | 2/30 | 0.20 (0.04 to 0.96) | 0.045 |
| Q4 | 9/82 | 1.38 (0.53 to 3.58) | 0.51 | 3/23 | 1.10 (0.22 to 5.45) | 0.91 | 3/30 | 0.30 (0.08 to 1.13) | 0.076 |
| CD163+ Quintile |  |  |  |  |  |  |  |  |  |
| Q1 | 6/66 | — |  | 3/19 | — |  | 8/24 | — |  |
| Q2 | 6/66 | 1.31 (0.42 to 4.07) | 0.64 | 1/19 | 0.36 (0.04 to 3.45) | 0.38 | 3/24 | 0.45 (0.12 to 1.68) | 0.23 |
| Q3 | 6/66 | 1.12 (0.36 to 3.48) | 0.84 | 2/18 | 0.85 (0.14 to 5.11) | 0.86 | 3/24 | 0.38 (0.10 to 1.44) | 0.15 |
| Q4 | 8/66 | 1.61 (0.56 to 4.66) | 0.38 | 1/18 | 0.56 (0.06 to 5.43) | 0.61 | 0/24 | 0.00 (0.00 to Inf) | >0.99 |
| Q5 | 8/66 | 1.68 (0.58 to 4.85) | 0.34 | 2/18 | 0.83 (0.14 to 4.97) | 0.84 | 3/24 | 0.32 (0.08 to 1.19) | 0.089 |
| Race |  |  |  |  |  |  |  |  |  |
| Black | 27/279 | — |  | 8/74 | — |  | 15/109 | — |  |
| White | 7/51 | 0.82 (0.34 to 1.96) | 0.66 | 1/18 | 0.20 (0.02 to 1.68) | 0.14 | 2/11 | 0.91 (0.20 to 4.09) | 0.90 |
| Age | 34/330 | 1.60 (1.30 to 1.96) | <0.001 | 9/92 | 1.69 (1.18 to 2.43) | 0.004 | 17/120 | 1.31 (1.05 to 1.64) | 0.016 |
| BMI | 34/329 | 1.39 (1.12 to 1.73) | 0.003 | 9/92 | 0.94 (0.48 to 1.87) | 0.87 | 17/120 | 0.84 (0.56 to 1.25) | 0.38 |
| Stage |  |  |  |  |  |  |  |  |  |
| I | 18/166 | — |  | 4/35 | — |  | 3/35 | — |  |
| II | 10/128 | 0.67 (0.31 to 1.46) | 0.31 | 4/52 | 0.73 (0.18 to 2.91) | 0.65 | 11/62 | 1.95 (0.54 to 7.02) | 0.31 |
| III/IV | 6/36 | 2.10 (0.83 to 5.29) | 0.12 | 1/5 | 3.25 (0.36 to 29.7) | 0.30 | 3/23 | 3.36 (0.67 to 16.9) | 0.14 |
| Grade |  |  |  |  |  |  |  |  |  |
| 3 | 12/110 | — |  | 5/58 | — |  | 15/103 | — |  |
| 1 | 7/68 | 0.88 (0.35 to 2.25) | 0.79 | 0/6 | 0.00 (0.00 to Inf) | >0.99 | 0/1 | 0.00 (0.00 to Inf) | >0.99 |
| 2 | 14/147 | 0.85 (0.39 to 1.84) | 0.68 | 3/27 | 1.40 (0.33 to 5.88) | 0.64 | 2/16 | 0.68 (0.16 to 2.99) | 0.61 |
| Tumor size | 32/328 | 1.12 (0.90 to 1.40) | 0.32 | 8/91 | 0.92 (0.49 to 1.74) | 0.80 | 17/119 | 1.09 (0.83 to 1.43) | 0.53 |
| Node status |  |  |  |  |  |  |  |  |  |
| Positive | 8/118 | — |  | 4/44 | — |  | 8/47 | — |  |
| Negative | 26/210 | 1.77 (0.80 to 3.90) | 0.16 | 5/47 | 0.95 (0.25 to 3.52) | 0.93 | 7/69 | 0.41 (0.15 to 1.14) | 0.087 |
| Surgery |  |  |  |  |  |  |  |  |  |
| No | 1/1 | — |  | 1/1 | — |  | 0/0 | — |  |
| Yes | 33/329 | 0.04 (0.01 to 0.30) | 0.002 | 8/91 | 0.03 (0.00 to 0.29) | 0.003 | 17/120 |  |  |
| Chemotherapy |  |  |  |  |  |  |  |  |  |
| No | 21/158 | — |  | 0/13 | — |  | 2/8 | — |  |
| Yes | 13/172 | 0.52 (0.26 to 1.04) | 0.065 | 9/79 |  |  | 15/112 | 0.47 (0.10 to 2.08) | 0.32 |
| Radiation |  |  |  |  |  |  |  |  |  |
| No | 8/102 | — |  | 4/39 | — |  | 6/29 | — |  |
| Yes | 26/228 | 1.56 (0.71 to 3.46) | 0.27 | 5/53 | 1.08 (0.29 to 4.03) | 0.91 | 11/91 | 0.66 (0.24 to 1.79) | 0.41 |
| Hormone therapy |  |  |  |  |  |  |  |  |  |
| No | 8/36 | — |  | 5/39 | — |  | 15/103 | — |  |
| Yes | 26/294 | 0.46 (0.21 to 1.01) | 0.053 | 4/53 | 0.51 (0.14 to 1.91) | 0.32 | 2/16 | 0.96 (0.22 to 4.24) | 0.96 |
| ^1^HR = Hazard Ratio, CI = Confidence Interval | | | | | | | | | |
| Age, BMI, and tumor size modeled continuously reflecting a 5-year increase in age, a 5-kg/m2 increase in BMI, and 1 centimeter increase in tumor size | | | | | | | | | |

Table S3. Multivariable Cox regression models estimating associations of CD163^+^ cell density tertiles with overall survival and breast cancer-specific survival within subtype, additionally adjusting for self-identified race and grade in Model 2

| Subtype, Model | | Overall Survival | | | Breast Cancer-Specific Survival | | |  |
| --- | --- | --- | --- | --- | --- | --- | --- | --- |
| Luminal | Characteristic | Events/N | HR (95% CI)^1^ | p-value | Events/N | HR (95% CI)^1^ | p-value |  |
| Model 1 | CD163+ |  |  |  |  |  |  |  |
|  | T1 | 24/110 | — |  | 10/110 | — |  |  |
|  | T2 | 17/110 | 0.81 (0.43 to 1.51) | 0.50 | 8/110 | 0.99 (0.39 to 2.52) | 0.98 |  |
|  | T3 | 24/110 | 0.99 (0.56 to 1.76) | 0.98 | 16/110 | 1.40 (0.63 to 3.13) | 0.41 |  |
|  | Age | 65/330 | 1.25 (1.10 to 1.42) | <0.001 | 34/330 | 1.56 (1.26 to 1.92) | <0.001 |  |
| Model 2 | CD163+ |  |  |  |  |  |  |  |
|  | T1 | 22/107 | — |  | 10/108 | — |  |  |
|  | T2 | 14/106 | 0.52 (0.26 to 1.05) | 0.068 | 7/107 | 0.86 (0.32 to 2.29) | 0.76 |  |
|  | T3 | 24/109 | 0.76 (0.41 to 1.43) | 0.40 | 16/109 | 1.23 (0.53 to 2.86) | 0.63 |  |
|  | Age | 60/322 | 1.25 (1.09 to 1.45) | 0.002 | 33/324 | 1.55 (1.24 to 1.94) | <0.001 |  |
|  | SIRE |  |  |  |  |  |  |  |
|  | Black | 46/273 | — |  | 27/275 | — |  |  |
|  | White | 14/49 | 1.39 (0.72 to 2.68) | 0.33 | 6/49 | 0.78 (0.31 to 1.99) | 0.60 |  |
|  | BMI | 60/322 | 1.28 (1.07 to 1.52) | 0.006 | 33/324 | 1.31 (1.04 to 1.65) | 0.022 |  |
|  | Grade |  |  |  |  |  |  |  |
|  | 1 | 10/68 | — |  | 7/68 | — |  |  |
|  | 2 | 22/144 | 1.06 (0.50 to 2.29) | 0.87 | 14/146 | 0.96 (0.38 to 2.41) | 0.93 |  |
|  | 3 | 28/110 | 1.59 (0.73 to 3.46) | 0.24 | 12/110 | 1.10 (0.42 to 2.93) | 0.84 |  |
|  | Stage |  |  |  |  |  |  |  |
|  | I | 26/165 | — |  |  |  |  |  |
|  | II | 17/126 | 0.52 (0.26 to 1.06) | 0.071 |  |  |  |  |
|  | III/IV | 17/31 | 2.57 (1.16 to 5.69) | 0.020 |  |  |  |  |
|  | Tumor size (cm) | 60/322 | 1.26 (1.09 to 1.46) | 0.002 |  |  |  |  |
| Subtype, Model | | Overall Survival | | | Breast Cancer-Specific Survival | | |  |
| HER2+ | Characteristic | Events/N | HR (95% CI)^1^ | p-value | Events/N | HR (95% CI)^1^ | p-value |  |
| Model 1 | CD163+ |  |  |  |  |  |  |  |
|  | T1 | 6/31 | — |  | 4/31 | — |  |  |
|  | T2 | 6/31 | 0.99 (0.31 to 3.15) | 0.99 | 2/31 | 0.42 (0.07 to 2.40) | 0.33 |  |
|  | T3 | 8/30 | 1.37 (0.47 to 4.04) | 0.57 | 3/30 | 0.54 (0.11 to 2.64) | 0.45 |  |
|  | Age | 20/92 | 1.26 (1.03 to 1.54) | 0.024 | 9/92 | 1.78 (1.21 to 2.61) | 0.003 |  |
| Model 2 | CD163+ |  |  |  |  |  |  |  |
|  | T1 | 6/31 | — |  | 4/31 | — |  |  |
|  | T2 | 6/31 | 0.74 (0.17 to 3.21) | 0.69 | 2/31 | 0.60 (0.08 to 4.38) | 0.61 |  |
|  | T3 | 7/29 | 0.84 (0.23 to 3.08) | 0.79 | 2/29 | 0.46 (0.07 to 3.18) | 0.43 |  |
|  | Age | 19/91 | 1.31 (1.04 to 1.63) | 0.019 | 8/91 | 1.70 (1.11 to 2.60) | 0.015 |  |
|  | SIRE |  |  |  |  |  |  |  |
|  | Black | 16/73 | — |  | 7/73 | — |  |  |
|  | White | 3/18 | 0.46 (0.11 to 1.90) | 0.28 | 1/18 | 0.36 (0.04 to 3.67) | 0.39 |  |
|  | Grade |  |  |  |  |  |  |  |
|  | 1/2 | 7/33 | — |  | 3/33 | — |  |  |
|  | 3 | 12/58 | 0.60 (0.19 to 1.90) | 0.39 | 5/58 | 0.58 (0.09 to 3.59) | 0.56 |  |
|  | Tumor size | 19/91 | 1.66 (1.09 to 2.53) | 0.018 |  |  |  |  |
| Subtype, Model | | Overall Survival | | | Breast Cancer-Specific Survival | | |  |
| Triple Negative | Characteristic | Events/N | HR (95% CI)^1^ | p-value | Events/N | HR (95% CI)^1^ | p-value |  |
| Model 1 | CD163+ |  |  |  |  |  |  |  |
|  | T1 | 20/40 | — |  | 9/40 | — |  |  |
|  | T2 | 12/40 | 0.59 (0.29 to 1.21) | 0.15 | 5/40 | 0.61 (0.20 to 1.85) | 0.38 |  |
|  | T3 | 8/40 | 0.36 (0.16 to 0.83) | 0.017 | 3/40 | 0.36 (0.10 to 1.38) | 0.14 |  |
|  | Age | 40/120 | 1.07 (0.93 to 1.23) | 0.34 | 17/120 | 1.26 (1.01 to 1.58) | 0.041 |  |
| Model 2 | CD163+ |  |  |  |  |  |  |  |
|  | T1 | 18/38 | — |  | 9/40 | — |  |  |
|  | T2 | 11/38 | 0.41 (0.18 to 0.95) | 0.037 | 5/40 | 0.54 (0.17 to 1.71) | 0.29 |  |
|  | T3 | 8/40 | 0.28 (0.11 to 0.69) | 0.006 | 3/40 | 0.30 (0.08 to 1.17) | 0.082 |  |
|  | Age | 37/116 | 1.29 (1.07 to 1.56) | 0.007 | 17/120 | 1.41 (1.09 to 1.83) | 0.009 |  |
|  | SIRE |  |  |  |  |  |  |  |
|  | Black | 33/105 | — |  | 15/109 | — |  |  |
|  | White | 4/11 | 0.51 (0.14 to 1.80) | 0.30 | 2/11 | 0.82 (0.15 to 4.42) | 0.82 |  |
|  | Grade |  |  |  |  |  |  |  |
|  | 1/2 | 5/17 | — |  | 2/17 | — |  |  |
|  | 3 | 32/99 | 2.25 (0.68 to 7.45) | 0.18 | 15/103 | 2.48 (0.43 to 14.4) | 0.31 |  |
|  | Stage |  |  |  |  |  |  |  |
|  | I | 5/34 | — |  | 3/35 | — |  |  |
|  | II | 17/60 | 1.48 (0.46 to 4.79) | 0.51 | 11/62 | 2.56 (0.66 to 9.93) | 0.17 |  |
|  | III/IV | 15/22 | 8.08 (2.06 to 31.7) | 0.003 | 3/23 | 7.01 (1.20 to 40.8) | 0.030 |  |
|  | Node status |  |  |  |  |  |  |  |
|  | Positive | 23/47 | — |  |  |  |  |  |
|  | Negative | 14/69 | 0.44 (0.18 to 1.08) | 0.074 |  |  |  |  |
| ^1^HR = Hazard Ratio, CI = Confidence Interval | | | | | | | |  |
| Age, BMI, and tumor size modeled continuously reflecting a 5-year increase in age, a 5-kg/m^2^ increase in BMI, and 1 centimeter increase in tumor size | | | | | | | |  |

Table S4. Multivariable Cox regression models assessing associations of additional CD163^+^ cell density cutoffs with overall survival within subtype

|  | | Model 1 | | | Model 2 | |
| --- | --- | --- | --- | --- | --- | --- |
| Subtype | CD163+ Density Cutoff | Events/N | HR (95% CI)^1^ | p-value | HR (95% CI)^1^ | p-value |
| Luminal | Median |  |  |  |  |  |
|  | Q1 | 37/165 | — |  | — |  |
|  | Q2 | 28/165 | 0.74 (0.45 to 1.22) | 0.24 | 0.61 (0.36 to 1.03) | 0.064 |
|  | Tertile |  |  |  |  |  |
|  | Q1 | 24/110 | — |  | — |  |
|  | Q2 | 17/110 | 0.81 (0.43 to 1.51) | 0.50 | 0.54 (0.28 to 1.07) | 0.076 |
|  | Q3 | 24/110 | 0.99 (0.56 to 1.76) | 0.98 | 0.83 (0.46 to 1.52) | 0.55 |
|  | Quartile |  |  |  |  |  |
|  | Q1 | 18/83 | — |  | — |  |
|  | Q2 | 19/83 | 1.32 (0.69 to 2.52) | 0.41 | 1.07 (0.54 to 2.09) | 0.85 |
|  | Q3 | 13/82 | 0.80 (0.39 to 1.63) | 0.54 | 0.55 (0.26 to 1.20) | 0.14 |
|  | Q4 | 15/82 | 0.91 (0.46 to 1.82) | 0.80 | 0.73 (0.35 to 1.50) | 0.39 |
|  | Quintile |  |  |  |  |  |
|  | Q1 | 13/66 | — |  | — |  |
|  | Q2 | 16/66 | 1.68 (0.80 to 3.52) | 0.17 | 1.61 (0.74 to 3.47) | 0.23 |
|  | Q3 | 11/66 | 1.08 (0.48 to 2.42) | 0.86 | 0.67 (0.28 to 1.65) | 0.39 |
|  | Q4 | 12/66 | 1.07 (0.49 to 2.36) | 0.86 | 0.83 (0.36 to 1.92) | 0.67 |
|  | Q5 | 13/66 | 1.17 (0.54 to 2.53) | 0.69 | 1.01 (0.44 to 2.28) | 0.99 |
| HER2+ | Median |  |  |  |  |  |
|  | Q1 | 7/46 | — |  | — |  |
|  | Q2 | 13/46 | 2.04 (0.80 to 5.20) | 0.14 | 1.57 (0.59 to 4.16) | 0.37 |
|  | Tertile |  |  |  |  |  |
|  | Q1 | 6/31 | — |  | — |  |
|  | Q2 | 6/31 | 0.99 (0.31 to 3.15) | 0.99 | 0.59 (0.16 to 2.16) | 0.43 |
|  | Q3 | 8/30 | 1.37 (0.47 to 4.04) | 0.57 | 0.67 (0.19 to 2.31) | 0.52 |
|  | Quartile |  |  |  |  |  |
|  | Q1 | 5/23 | — |  | — |  |
|  | Q2 | 2/23 | 0.38 (0.07 to 1.98) | 0.25 | 0.20 (0.04 to 1.18) | 0.075 |
|  | Q3 | 7/23 | 1.59 (0.48 to 5.30) | 0.45 | 1.03 (0.26 to 4.10) | 0.97 |
|  | Q4 | 6/23 | 1.22 (0.37 to 4.04) | 0.75 | 0.50 (0.12 to 2.02) | 0.33 |
|  | Quintile |  |  |  |  |  |
|  | Q1 | 5/19 | — |  | — |  |
|  | Q2 | 1/19 | 0.23 (0.03 to 1.98) | 0.18 | 0.12 (0.01 to 1.10) | 0.061 |
|  | Q3 | 5/18 | 0.99 (0.28 to 3.51) | >0.99 | 0.67 (0.17 to 2.59) | 0.56 |
|  | Q4 | 4/18 | 1.01 (0.26 to 3.91) | >0.99 | 0.42 (0.09 to 2.10) | 0.29 |
|  | Q5 | 5/18 | 1.16 (0.33 to 4.02) | 0.82 | 0.45 (0.10 to 1.97) | 0.29 |
| Triple Negative | Median |  |  |  |  |  |
|  | Q1 | 27/60 | — |  | — |  |
|  | Q2 | 13/60 | 0.43 (0.22 to 0.86) | 0.017 | 0.58 (0.28 to 1.22) | 0.15 |
|  | Tertile |  |  |  |  |  |
|  | Q1 | 20/40 | — |  | — |  |
|  | Q2 | 12/40 | 0.59 (0.29 to 1.21) | 0.15 | 0.44 (0.19 to 1.02) | 0.056 |
|  | Q3 | 8/40 | 0.36 (0.16 to 0.83) | 0.017 | 0.29 (0.12 to 0.73) | 0.008 |
|  | Quartile |  |  |  |  |  |
|  | Q1 | 17/30 | — |  | — |  |
|  | Q2 | 10/30 | 0.58 (0.26 to 1.26) | 0.17 | 0.24 (0.09 to 0.63) | 0.004 |
|  | Q3 | 6/30 | 0.32 (0.12 to 0.82) | 0.018 | 0.38 (0.14 to 1.01) | 0.053 |
|  | Q4 | 7/30 | 0.36 (0.15 to 0.89) | 0.027 | 0.27 (0.10 to 0.70) | 0.007 |
|  | Quintile |  |  |  |  |  |
|  | Q1 | 14/24 | — |  | — |  |
|  | Q2 | 9/24 | 0.76 (0.33 to 1.75) | 0.52 | 0.30 (0.10 to 0.85) | 0.024 |
|  | Q3 | 6/24 | 0.43 (0.17 to 1.13) | 0.086 | 0.17 (0.06 to 0.51) | 0.002 |
|  | Q4 | 6/24 | 0.46 (0.17 to 1.23) | 0.12 | 0.30 (0.11 to 0.85) | 0.024 |
|  | Q5 | 5/24 | 0.33 (0.12 to 0.93) | 0.037 | 0.15 (0.05 to 0.48) | 0.001 |
| ^1^HR = Hazard Ratio, CI = Confidence Interval | | | | | | |
| Model 1 adjusted for age across all subtypes. In Luminal, Model 2 adjusted for age, BMI, stage, and tumor size. In HER2+, Model 2 adjusted for age and tumor size. In Triple Negative, Model 2 adjusted for age, stage, grade, and lymph node status | | | | | | |

Table S5. Multivariable Cox regression models assessing associations of additional CD163^+^ cell density cutoffs with breast cancer-specific survival within subtype

|  | | Model 1 | | | Model 2 | |
| --- | --- | --- | --- | --- | --- | --- |
| Subtype | Characteristic | Events/N | HR (95% CI)^1^ | p-value | HR (95% CI)^1^ | p-value |
| Luminal | Median |  |  |  |  |  |
|  | Q1 | 16/165 | — |  | — |  |
|  | Q2 | 18/165 | 1.02 (0.52 to 2.02) | 0.94 | 0.94 (0.48 to 1.87) | 0.87 |
|  | Tertile |  |  |  |  |  |
|  | Q1 | 10/110 | — |  | — |  |
|  | Q2 | 8/110 | 0.99 (0.39 to 2.52) | 0.98 | 0.99 (0.39 to 2.52) | 0.98 |
|  | Q3 | 16/110 | 1.40 (0.63 to 3.13) | 0.41 | 1.27 (0.57 to 2.86) | 0.56 |
|  | Quartile |  |  |  |  |  |
|  | Q1 | 8/83 | — |  | — |  |
|  | Q2 | 8/83 | 1.31 (0.49 to 3.51) | 0.59 | 1.30 (0.48 to 3.48) | 0.61 |
|  | Q3 | 9/82 | 1.27 (0.49 to 3.30) | 0.63 | 1.11 (0.42 to 2.93) | 0.83 |
|  | Q4 | 9/82 | 1.07 (0.41 to 2.83) | 0.88 | 1.03 (0.39 to 2.72) | 0.95 |
|  | Quintile |  |  |  |  |  |
|  | Q1 | 6/66 | — |  | — |  |
|  | Q2 | 6/66 | 1.58 (0.50 to 4.94) | 0.44 | 1.57 (0.50 to 4.92) | 0.44 |
|  | Q3 | 6/66 | 1.43 (0.46 to 4.46) | 0.54 | 1.53 (0.49 to 4.78) | 0.47 |
|  | Q4 | 8/66 | 1.57 (0.54 to 4.55) | 0.41 | 1.23 (0.41 to 3.67) | 0.72 |
|  | Q5 | 8/66 | 1.40 (0.48 to 4.10) | 0.54 | 1.38 (0.47 to 4.04) | 0.55 |
| HER2+ | Median |  |  |  |  |  |
|  | Q1 | 5/46 | — |  | — |  |
|  | Q2 | 4/46 | 0.67 (0.17 to 2.67) | 0.57 |  |  |
|  | Tertile |  |  |  |  |  |
|  | Q1 | 4/31 | — |  | — |  |
|  | Q2 | 2/31 | 0.42 (0.07 to 2.40) | 0.33 |  |  |
|  | Q3 | 3/30 | 0.54 (0.11 to 2.64) | 0.45 |  |  |
|  | Quartile |  |  |  |  |  |
|  | Q1 | 3/23 | — |  | — |  |
|  | Q2 | 2/23 | 0.60 (0.10 to 3.69) | 0.59 |  |  |
|  | Q3 | 1/23 | 0.26 (0.02 to 2.82) | 0.27 |  |  |
|  | Q4 | 3/23 | 0.75 (0.14 to 3.92) | 0.74 |  |  |
|  | Quintile |  |  |  |  |  |
|  | Q1 | 3/19 | — |  | — |  |
|  | Q2 | 1/19 | 0.50 (0.05 to 4.92) | 0.55 |  |  |
|  | Q3 | 2/18 | 0.49 (0.08 to 3.13) | 0.45 |  |  |
|  | Q4 | 1/18 | 0.29 (0.03 to 3.37) | 0.33 |  |  |
|  | Q5 | 2/18 | 0.68 (0.11 to 4.14) | 0.67 |  |  |
| Triple Negative | Median |  |  |  |  |  |
|  | Q1 | 12/60 | — |  | — |  |
|  | Q2 | 5/60 | 0.45 (0.15 to 1.33) | 0.15 | 0.52 (0.17 to 1.54) | 0.24 |
|  | Tertile |  |  |  |  |  |
|  | Q1 | 9/40 | — |  | — |  |
|  | Q2 | 5/40 | 0.61 (0.20 to 1.85) | 0.38 | 0.70 (0.23 to 2.13) | 0.53 |
|  | Q3 | 3/40 | 0.36 (0.10 to 1.38) | 0.14 | 0.38 (0.10 to 1.44) | 0.15 |
|  | Quartile |  |  |  |  |  |
|  | Q1 | 8/30 | — |  | — |  |
|  | Q2 | 4/30 | 0.48 (0.14 to 1.60) | 0.23 | 0.37 (0.11 to 1.29) | 0.12 |
|  | Q3 | 2/30 | 0.27 (0.06 to 1.33) | 0.11 | 0.33 (0.07 to 1.64) | 0.17 |
|  | Q4 | 3/30 | 0.38 (0.10 to 1.48) | 0.16 | 0.35 (0.09 to 1.35) | 0.13 |
|  | Quintile |  |  |  |  |  |
|  | Q1 | 8/24 | — |  | — |  |
|  | Q2 | 3/24 | 0.43 (0.11 to 1.61) | 0.21 | 0.32 (0.08 to 1.24) | 0.10 |
|  | Q3 | 3/24 | 0.42 (0.11 to 1.62) | 0.21 | 0.37 (0.10 to 1.45) | 0.16 |
|  | Q4 | 0/24 | 0.00 (0.00 to Inf) | >0.99 | 0.00 (0.00 to Inf) | >0.99 |
|  | Q5 | 3/24 | 0.39 (0.10 to 1.50) | 0.17 | 0.33 (0.08 to 1.29) | 0.11 |
| ^1^HR = Hazard Ratio, CI = Confidence Interval | | | | | | |
| Model 1 adjusted for age across all subtypes. In Luminal, Model 2 adjusted for age and BMI. In Triple Negative, Model 2 adjusted for age and stage | | | | | | |

Table S6. Multivariable Cox regression models assessing associations between CD163^+^ cell density tertiles with overall survival and breast cancer-specific survival by ER status

| Overall Survival | | ER+ | | | ER- | | |
| --- | --- | --- | --- | --- | --- | --- | --- |
| Model | Characteristic | Events/N | HR (95% CI)^1^ | p-value | Events/N | HR (95% CI)^1^ | p-value |
| Model 1 | CD163+ |  |  |  |  |  |  |
|  | T1 | 28/129 | — |  | 24/54 | — |  |
|  | T2 | 19/128 | 0.75 (0.42 to 1.34) | 0.33 | 18/54 | 0.73 (0.40 to 1.35) | 0.31 |
|  | T3 | 27/128 | 1.02 (0.60 to 1.75) | 0.93 | 11/53 | 0.44 (0.22 to 0.91) | 0.027 |
|  | Age | 74/385 | 1.26 (1.12 to 1.42) | <0.001 | 53/161 | 1.08 (0.96 to 1.22) | 0.20 |
| Model 2 | CD163+ |  |  |  |  |  |  |
|  | T1 | 27/128 | — |  | 24/54 | — |  |
|  | T2 | 17/126 | 0.53 (0.29 to 1.00) | 0.048 | 18/54 | 0.73 (0.40 to 1.36) | 0.33 |
|  | T3 | 26/127 | 0.85 (0.49 to 1.50) | 0.58 | 11/53 | 0.39 (0.19 to 0.80) | 0.011 |
|  | Age | 70/381 | 1.29 (1.13 to 1.46) | <0.001 | 53/161 | 1.18 (1.04 to 1.35) | 0.011 |
|  | BMI | 70/381 | 1.22 (1.03 to 1.44) | 0.018 |  |  |  |
|  | Stage |  |  |  |  |  |  |
|  | I | 28/187 | — |  | 8/49 | — |  |
|  | II | 22/158 | 0.70 (0.38 to 1.29) | 0.25 | 27/85 | 2.53 (1.13 to 5.65) | 0.024 |
|  | III/IV | 20/36 | 3.65 (1.86 to 7.17) | <0.001 | 18/27 | 11.4 (4.76 to 27.4) | <0.001 |
|  | Tumor size | 70/381 | 1.25 (1.10 to 1.42) | <0.001 |  |  |  |
| Breast Cancer-Specific Survival | | ER+ | | | ER- | | |
| Model | Characteristic | Events/N | HR (95% CI)^1^ | p-value | Events/N | HR (95% CI)^1^ | p-value |
| Model 1 | CD163+ |  |  |  |  |  |  |
|  | T1 | 12/129 | — |  | 12/54 | — |  |
|  | T2 | 10/128 | 0.96 (0.41 to 2.23) | 0.92 | 9/54 | 0.75 (0.31 to 1.78) | 0.51 |
|  | T3 | 15/128 | 1.19 (0.55 to 2.58) | 0.66 | 3/53 | 0.27 (0.08 to 0.98) | 0.046 |
|  | Age | 37/385 | 1.59 (1.31 to 1.93) | <0.001 | 24/161 | 1.28 (1.06 to 1.54) | 0.009 |
| Model 2 | CD163+ |  |  |  |  |  |  |
|  | T1 | 12/129 | — |  | 12/54 | — |  |
|  | T2 | 10/127 | 0.89 (0.38 to 2.08) | 0.79 | 9/54 | 0.69 (0.28 to 1.65) | 0.40 |
|  | T3 | 15/128 | 1.01 (0.46 to 2.24) | 0.97 | 3/53 | 0.24 (0.07 to 0.86) | 0.028 |
|  | Age | 37/384 | 1.57 (1.28 to 1.92) | <0.001 | 24/161 | 1.37 (1.12 to 1.67) | 0.002 |
|  | BMI | 37/384 | 1.35 (1.08 to 1.70) | 0.009 |  |  |  |
|  | Stage |  |  |  |  |  |  |
|  | I | 20/187 | — |  | 5/49 | — |  |
|  | II | 10/158 | 0.67 (0.31 to 1.43) | 0.30 | 16/85 | 2.80 (0.99 to 7.92) | 0.052 |
|  | III/IV | 7/39 | 2.96 (1.22 to 7.16) | 0.016 | 3/27 | 4.41 (0.98 to 19.8) | 0.052 |
| ^1^HR = Hazard Ratio, CI = Confidence Interval | | | | | | | |
| Age, BMI, and tumor size modeled continuously reflecting a 5-year increase in age, a 5-kg/m2 increase in BMI, and 1 centimeter increase in tumor size | | | | | | | |

Table S7. Multivariable Cox regression models assessing associations between CD163^+^ cell density tertiles with overall survival and breast cancer-specific survival within Black cases

|  | | Overall Survival | | | Breast Cancer-Specific Survival | | |
| --- | --- | --- | --- | --- | --- | --- | --- |
| Model | Characteristic | Events/N | HR (95% CI)^1^ | p-value | Events/N | HR (95% CI)^1^ | p-value |
| Model 1 | CD163+ |  |  |  |  |  |  |
|  | T1 | 42/156 | — |  | 22/156 | — |  |
|  | T2 | 31/155 | 0.77 (0.48 to 1.23) | 0.27 | 15/155 | 0.71 (0.37 to 1.37) | 0.31 |
|  | T3 | 32/155 | 0.83 (0.52 to 1.32) | 0.43 | 14/155 | 0.71 (0.36 to 1.39) | 0.32 |
|  | Age | 105/466 | 1.13 (1.03 to 1.24) | 0.007 | 51/466 | 1.42 (1.22 to 1.65) | <0.001 |
| Model 2 | CD163+ |  |  |  |  |  |  |
|  | T1 | 40/154 | — |  | 22/155 | — |  |
|  | T2 | 27/149 | 0.60 (0.36 to 0.99) | 0.045 | 14/152 | 0.61 (0.31 to 1.22) | 0.16 |
|  | T3 | 31/154 | 0.54 (0.33 to 0.91) | 0.019 | 14/155 | 0.51 (0.25 to 1.05) | 0.067 |
|  | Age | 98/457 | 1.27 (1.14 to 1.40) | <0.001 | 50/462 | 1.49 (1.28 to 1.74) | <0.001 |
|  | BMI | 98/457 | 1.14 (0.98 to 1.31) | 0.083 |  | — |  |
|  | Stage |  |  |  |  |  |  |
|  | I | 31/203 | — |  | 22/203 | — |  |
|  | II | 39/204 | 0.91 (0.53 to 1.53) | 0.71 | 20/206 | 1.07 (0.57 to 2.00) | 0.83 |
|  | III/IV | 28/50 | 3.76 (1.94 to 7.30) | <0.001 | 8/53 | 3.34 (1.44 to 7.74) | 0.005 |
|  | Subtype |  |  |  |  |  |  |
|  | Luminal | 47/276 | — |  | 27/279 | — |  |
|  | Triple Negative | 35/108 | 2.96 (1.82 to 4.81) | <0.001 | 15/109 | 2.80 (1.42 to 5.51) | 0.003 |
|  | HER2+ | 16/73 | 2.01 (1.10 to 3.65) | 0.023 | 8/74 | 1.39 (0.62 to 3.12) | 0.43 |
|  | Tumor size (cm) | 98/457 | 1.22 (1.09 to 1.37) | <0.001 |  | — |  |
| ^1^HR = Hazard Ratio, CI = Confidence Interval | | | | | | | |
| Age, BMI, and tumor size modeled continuously reflecting a 5-year increase in age, a 5-kg/m2 increase in BMI, and 1 centimeter increase in tumor size | | | | | | | |

Figure S1. Diagram of participant availability for CD163 profiling in the Women’s Circle of Health Study.


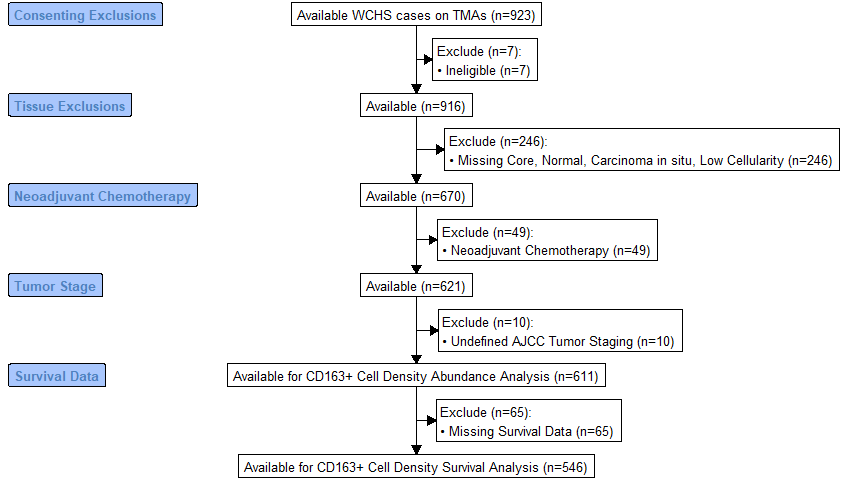


**Supplemental Methods**

**Study population**

The data and tumor specimens for this study were drawn from the Women’s Circle of Health (WCHS),^1^ a case-control study designed to examine risk factors for aggressive breast cancer in Black and White women, and the Women’s Circle of Health Follow-up Study (WCHFS),^2^ which expanded the scope of WCHS to include factors that affect survivorship and prognosis.

Women in WCHS were diagnosed between 2001 and 2013. Initially, women were recruited from metropolitan New York City (NYC), working with hospitals that had the greatest referral patterns for Black women in the boroughs of Manhattan, Brooklyn, Queens, and the Bronx. Since 2006, the New Jersey State Cancer Registry (NJSCR), a Surveillance, Epidemiology and End Results Program (SEER) site, identified eligible patients from 10 counties in NJ, using rapid case ascertainment. Recruitment in metropolitan NYC was subsequently phased out.

WCHFS is a longitudinal cohort study of Black breast cancer survivors diagnosed between 2013 and 2019, built upon the infrastructure and methodology of WCHS. WCHFS expanded recruitment in the same counties in New Jersey and broadened the scope to evaluate factors potentially affecting survivorship and prognosis. WCHFS also includes annual linkages with NJSCR files for survival outcomes for all Black cases recruited in WCHS and WCHFS in New Jersey.

For both WCHS and WCHFS, participants were 20–75 years old; self-identified as Black or White; had primary, histologically confirmed invasive breast cancer or ductal carcinoma in situ (DCIS); and had no previous history of cancer other than non-melanoma skin cancer. Upon consent, an in-depth at-home interview was conducted to collect data on established and suspected risk factors for breast cancer, and cases signed a release for their formalin-fixed paraffin-embedded (FFPE) tumor blocks and medical records. For WCHFS, detailed information was also collected on survivorship factors, such as access to care, patient reported outcomes, obesity-related comorbidities and their management, health related quality of life, lifestyle and social determinants of health, medication adherence, new breast cancer events, and medical and pharmacy records.

Tumors from all patients with enough tumor tissue were used for construction of tissue microarrays (TMAs), with each TMA containing at least two 0.6mm cores. At the time this study was initiated, cases diagnosed from 2001-2017 were built into TMAs.

**Immunohistochemical staining and image analysis**

*IHC Staining*

Slides were added to the Leica Bond Rx, where they were deparaffinized with Bond Dewax Solution (Leica AR9222) and rinsed in water. Bond Epitope Retrieval 2 (Leica AR9640) was used for target retrieval for 30 minutes. Slides were blocked using peroxide block from Bond Polymer Refine Detection kit (Leica DS9800) for 5 minutes. Slides were incubated with CD163 primary antibody (Leica-Novocastra NCL-CD163) for 20 mins at 1/900 dilution. The Bond Polymer Refine Detection kit (Leica DS9800) reagents were used as follows: Post Primary was applied for 8 minutes, followed by Polymer for 8 minutes, and then DAB (Diaminobenzidine) for 10 minutes. Slides were counterstained with Hematoxylin for 8 minutes then placed into water. After removing slides from the Leica Bond Rx they were dehydrated, cleared, and coverslipped.

*Aperio Slide Scanning and Image Analysis*

TMA slides stained for CD163 were digitally scanned using Aperio AT2 (Leica Biosystems, Inc., Buffalo Grove, IL) with 20x bright-field microscopy. These images are then accessible using eSlide Manager (Leica Biosystems, Inc., Buffalo Grove, IL), a web-based digital pathology information management system. Slide images are automatically associated to a digital slide created in the Digital Slide table in Spectrum. Once slides are scanned, Aperio ImageScope version 12.4.3.8007 (Leica Biosystems, Inc., Buffalo Grove, IL) was used to view images for analysis. Slide image data fields were populated, images were examined for quality and amended as necessary. An annotation layer was created for each core of interest in the TMA.

The Aperio image analysis platform was used to develop a quantitative image analysis algorithm macro for the quantification of CD163 stain. These algorithms used color de‐convolution to separate diaminobenzidine (DAB) from the hematoxylin counterstain thereby providing stain separation. An algorithm was used to analyze DAB staining and the percentage of cells containing stain. The algorithm results included total number of cells, percentage of positive and negative cells, and the area of tissue analyzed (mm^2^). Image analysis data was exported from Spectrum as a .csv file and converted to a .xls file and formatted using Microsoft Excel. The number of CD163 positive cells was reported per square millimeter using a simple formula in excel to calculate the final variable – cell density.

**References**

1. Ambrosone CB, Ciupak GL, Bandera EV, et al. Conducting Molecular Epidemiological Research in the Age of HIPAA: A Multi-Institutional Case-Control Study of Breast Cancer in African-American and European-American Women. J Oncol 2009;2009:871250.

2. Bandera EV, Demissie K, Qin B, et al. The Women's Circle of Health Follow-Up Study: a population-based longitudinal study of Black breast cancer survivors in New Jersey. J Cancer Surviv 2020;14:331-46.
